# Consumer Views on Using Digital Data for COVID-19 Control in the United States

**DOI:** 10.1001/jamanetworkopen.2021.10918

**Published:** 2021-05-19

**Authors:** David Grande, Nandita Mitra, Xochitl Luna Marti, Raina Merchant, David Asch, Abby Dolan, Meghana Sharma, Carolyn Cannuscio

**Affiliations:** 1 Leonard Davis Institute of Health Economics, University of Pennsylvania, Philadelphia; 2 Division of General Internal Medicine, University of Pennsylvania, Philadelphia; 3 Department of Biostatistics, Epidemiology and Informatics, University of Pennsylvania, Philadelphia; 4 Department of Emergency Medicine, University of Pennsylvania, Philadelphia; 5 Center for Digital Health, University of Pennsylvania, Philadelphia; 6 Penn Medicine Center for Health Care Innovation, University of Pennsylvania, Philadelphia; 7 Department of Family Medicine and Community Health, University of Pennsylvania, Philadelphia

## Abstract

**Importance:**

Curbing COVID-19 transmission is currently the greatest global public health challenge. Consumer digital tools used to collect data, such as the Apple-Google digital contact tracing program, offer opportunities to reduce COVID-19 transmission but introduce privacy concerns.

**Objective:**

To assess uses of consumer digital information for COVID-19 control that US adults find acceptable and the factors associated with higher or lower approval of use of this information.

**Design, Setting, and Participants:**

This cross-sectional survey study obtained data from a nationally representative sample of 6284 US adults recruited by email from the web-based Ipsos KnowledgePanel in July 2020. Respondents evaluated scenarios reflecting uses of digital data for COVID-19 control (case identification, digital contact tracing, policy setting, and enforcement of quarantines).

**Main Outcomes and Measures:**

Levels of support for use of personal digital data in 9 scenarios to mitigate the spread of COVID-19 infection, rated on a Likert scale, ranging from 1 (strongly disagree) to 5 (strongly agree). Multivariable linear regression models were fitted for each scenario and included factors hypothesized to be associated with views about digital data use for COVID-19 mitigation measures. Black and Hispanic survey respondents were oversampled; thus, poststratification weights were used so that results are representative of the general US population.

**Results:**

Of 6284 individuals invited to participate in the study, 3547 responded, for a completion rate of 56%. A total of 1762 participants (52%) were female, 715 (21%) identified as Black, 790 (23%) identified as Hispanic, and 1224 (36%) were 60 years or older; mean (SD) age was 51.7 (16.6) years. Approval of scenarios was low, ranging from 28% to 43% (52%-67% when neutral responses were included). Differences were found based on digital data source (smartphone vs social media: coefficient, 0.29 [95% CI, 0.23-0.35]; *P* < .001; smart thermometer vs social media: coefficient, 0.09 [95% CI, 0.03-0.16]; *P* = .004). County COVID-19 rates (coefficient, −0.02; 95% CI, −0.16 to 0.13 for quartile 4 compared with quartile 1) and prior family diagnosis of COVID-19 (coefficient, 0.00; 95% CI, −0.25 to 0.25) were not associated with support. Compared with self-described liberal individuals, conservative (coefficient, −0.81; 95% CI, −0.96 to −0.66; *P* < .001) and moderate (coefficient, −0.52; 95% CI, −0.67 to −0.38; *P* < .001) individuals were less likely to support the scenarios. Similarly, large political differences were observed in support of the Apple-Google digital contact tracing program, with less support from conservative (coefficient, −0.99; 95% CI, −1.11 to −0.87; *P* < .001) and moderate (coefficient, −0.59; 95% CI, −0.69 to −0.48; *P* < .001) individuals compared with liberal individuals. Respondents from racial/ethnic minority groups were more supportive of the scenarios than were White, non-Hispanic respondents. For example, compared with White respondents, Black respondents were more supportive of the Apple-Google contact tracing program (coefficient, 0.20; 95% CI, 0.07-0.32; *P* = .002).

**Conclusions and Relevance:**

In this survey study of US adults, many were averse to their information being used on digital platforms to mitigate transmission of COVID-19. These findings suggest that in current and future pandemics, public health departments should use multiple strategies to gain public trust and accelerate adoption of tools such as digital contact tracing applications.

## Key Points

**Question:**

What uses of consumer digital information for COVID-19 control are most acceptable to US adults, and what factors are associated with higher or lower approval of use of this information?

**Findings:**

In this cross-sectional survey study of 6284 US adults, approval was generally low for use of consumer digital data for activities such as case identification, digital contact tracing, policy setting, and enforcing quarantines. Political ideology and race/ethnicity were associated with approval for scenarios in which digital data were used, whereas local COVID-19 incidence and family experience with COVID were not.

**Meaning:**

The findings suggest that in current and future pandemics, public health departments should use multiple strategies to gain public trust and accelerate adoption of tools such as digital contact tracing applications.

## Teaser

This cross-sectional survey study assesses use of consumer digital information for COVID-19 control that US adults consider to be acceptable and the factors associated with higher or lower approval of use of this information.

## Introduction

The COVID-19 pandemic has prompted governments around the world to use digital information in novel ways as a public health intervention to decrease the transmission of disease.^1,2,3^ The Chinese government tracked population movement around the country with closed circuit television and geolocating wristbands.^4^ South Korea quickly brought the COVID-19 outbreak under control through a rapid testing campaign driven by data from credit card transactions, smartphone location tracking, and closed circuit television.^5^ In the US, internet-connected devices such as smart thermometers have been used to track the geographic distribution of influenza-like symptoms, and smartphones have been used to measure population mobility in response to various public health restrictions.^6,7,8^

More recently, public health departments have used new functionality in the Apple and Google smartphone operating systems to deploy digital contact tracing programs. Bluetooth measures proximity to other smartphones and alerts program users if they have come into close contact with a person who tested positive for SARS-CoV-2.^9^ Apple and Google have prioritized privacy; data sharing with public health agencies is opt in, meaning that the consumer must choose to turn it on. There is concern that these privacy protections could lead to low rates of participation, which could render the program ineffective.^10,11,12^

The COVID-19 pandemic has led to the initiation of other public health–related uses of consumer digital information focused on disease surveillance, contact tracing, and enforcement of measures such as quarantines.^13,14^ Use of digital information for COVID-19 response has raised new ethical considerations and questions about how consumers view the tradeoff between public health and individual privacy. On one hand, digital technology has the potential to markedly accelerate public health measures that can decrease chains of disease transmission. On the other hand, privacy advocates have questioned how personal data will be protected, retained, and deidentified.^15,16^ In the process of designing programs and policies, debates have often focused on whether participation should be voluntary (ie, opt in vs opt out), whether data should be reported and acted on at the individual or population level, and whether digital data should be used for enforcement of public health measures.^17^ Some data use, such as the Apple-Google Bluetooth-enabled digital contact tracing program, requires a high level of participation to be effective. Polling in April 2020^18^ by the Kaiser Family Foundation showed that 45% of respondents were willing to download and use a contact tracing application, and individuals in areas with higher COVID-19 rates were more willing to do so than were those in areas with lower rates.

In this nationally representative survey study, we assessed consumers’ attitudes about 9 use cases for personal digital information to mitigate the impact of the COVID-19 pandemic. We included versions of the Apple-Google digital contact tracing program that is currently being deployed in many US states. We hypothesized that views would vary based on several factors, including political ideology (given partisan debates about COVID-19 policies), the potential for personal and community benefit (local rates, belonging to a population disproportionately affected by COVID-19), a family history of COVID-19, and whether use cases were written to describe individual-level or population-level data use. Understanding consumer views on digital privacy is critical as the world confronts the immediate challenges of the COVID-19 pandemic and considers preparedness for future pandemics.

## Methods

### Participants

This cross-sectional survey study used responses to a survey administered from July 10 through July 31, 2020. Participants were recruited from the web-enabled Ipsos KnowledgePanel, a probability-based panel designed to be representative of the US population.^19^ At the time they joined the panel, respondents completed a general informed consent process through an online survey during the KnowledgePanel recruitment process. All data received by the study team were deidentified. Ipsos KnowledgePanel provided the study team with the following measures reflective of the recruitment of individuals into the panel: 11.5% average recruitment rate, 59.5% average household profile completion rate, and 36.2% average household retention rate. This study was reviewed and deemed exempt by the institutional review board at the University of Pennsylvania based on the minimal risk of the research and use of deidentified data. This study followed the American Association for Public Opinion Research (AAPOR) reporting guideline.

Race/ethnicity was assessed in this study because prior evidence has shown elevated concern regarding digital health privacy among historically oppressed racial/ethnic groups.^20,21^ When participants joined the panel, they were asked to complete a core survey profile, which included questions about their racial/ethnic background using the US Census Bureau categories and questions. In addition, we oversampled Black and Hispanic participants given the disproportionate effects of COVID-19 in these populations to allow for comparisons by race/ethnicity.^22,23^ The survey was administered in both Spanish and English.

### Survey Development and Administration

This study reports responses to scenarios involving the use of consumer digital information to mitigate the spread of COVID-19. The scenarios were developed based on actual or possible data applications described in academic literature, the lay press, and white papers.^6,24,25,26^ The scenarios varied in 3 general dimensions: the source of digital data (social media, smart thermometer, or smartphone), the sharing of data with public health officials, and the particular use. The scenarios are described in eAppendix 1 in the Supplement and summarized in the following paragraph.

Scenarios 1 and 2 described using social media data (online chatter) to monitor for COVID-19–related symptoms. Scenario 1 described using aggregate data to set public health policies. Scenario 2 described using the data to contact symptomatic individuals for COVID-19 testing. Scenarios 3 and 4 were analogous to scenarios 1 and 2, but the data source was body temperature readings from smart thermometers. Scenarios 5, 6, and 7 described versions of the Apple-Google digital contact tracing program (scenario 5: opt in, optional data sharing with public health agencies; scenario 6: opt in, automatic data sharing with public health agencies; scenario 7: automatic participation, automatic data sharing with public health agencies). The Apple-Google digital contact tracing program, actively deployed by many health departments, uses Bluetooth technology to measure exposure (time/distance) to other people. If an individual has a positive test result, the program will notify individuals enrolled in the program who are found to be close contacts of the COVID-19–positive individual. To date, the program is voluntary and does not share data with public health authorities. Scenario 8 described using smartphones to enforce quarantines. Scenario 9 described developing COVID-19 risk profiles on smartphones that could be used to restrict certain activities.

We randomly assigned participants to receive questions framed at the individual level vs questions framed at the population level to assess whether individual-level saliency influenced responses. Those in the individual-level framing arm of the study responded to scenarios emphasizing use of their own data (eg, “your” data). Those in the population-level framing arm responded to scenarios emphasizing the use of data more generally (eg, “people’s” data). We hypothesized that support for scenarios would be greater for those in the population-level framing arm because the individual-level framing arm would highlight individual privacy concerns.

For each scenario, participants rated their approval on a Likert scale with the following options: strongly disagree (1), disagree (2), neither agree nor disagree (3), agree (4), or strongly agree (5). The content of other questions included self-reported political ideology and overall health status and whether the respondent or a family member had been diagnosed with COVID-19. Sociodemographic data were previously collected by Ipsos and shared with the study team.

### Statistical Analysis

We report study-specific survey completion rates as recommended for online probability samples. Panel recruitment and retention rates were reported previously.^27^ Descriptive statistics, including means and 95% CIs, were calculated for each scenario. Because we oversampled Black and Hispanic survey respondents, poststratification weights were used so that results are representative of the general US population. Of the survey respondents, 158 (4%) had incomplete data. All variables used to develop poststratification weights were complete because they were collected at the time of participants’ entry to the panel. All other variables (COVID-19 scenarios, political ideology, and personal or family history of COVID-19) were more than 98% complete. We used multiple imputation by chained equations and compared the results with results of a complete case analysis and those of an extreme case analysis. The results were nearly identical (eAppendixes 2 and 3 in the Supplement).

We fit multivariable linear regression models for each scenario. We included factors that we hypothesized would influence views about digital data use for COVID-19 mitigation measures: political ideology and geography, prior personal or family experience with COVID-19, sociodemographic factors associated with COVID-19 risk (race/ethnicity, household income), and county-level COVID-19 rates. We retrieved COVID-19 case counts and rates from usafacts.org for a 2-week period during survey administration (July 8-22, 2020) and linked them to a respondent’s county of residence.

We then conducted a factor analysis to group similar scenarios for data reduction and better interpretability. Factor loadings revealed 3 factors among the 9 items with eigenvalues above 1 (*P* < .001): (1) social media data for identifying cases (scenarios 1 and 2); (2) smart thermometer data for identifying cases (scenarios 3 and 4); (3) smartphone data for contact tracing, quarantining, and risk-stratifying individuals (scenarios 5-9). We analyzed the 3 factors together using a multivariable generalized estimating equation model to account for correlation of responses from a participant. We assumed a linear mean model, independent working correlation structure, and empirical SEs. The final model included these 3 factors along with the experimental arm from the framing experiment (individual-level vs population-level framing) and the covariates described above from scenario-specific models. Stata Statistical Software, version 16.1 (StataCorp LLC) was used for all corresponding analyses. A 2-tailed *P* = .05 was considered to be statistically significant.

## Results

Of 6284 recruited individuals, 3547 responded to the survey, representing a completion rate of 56%. This included 158 individuals who did not respond to all 9 scenarios but whose data were imputed and therefore included in multivariable analyses. Table 1 describes the characteristics of the survey respondents. A total of 1762 participants (52%) were female, 715 (21%) identified as Black, 790 (23%) identified as Hispanic, and 1224 (36%) were 60 years or older; mean (SD) age was 51.7 (16.6) years. A total of 960 individuals (28%) were non-White, reflecting oversampling of minority populations.

**Table 1.** Characteristics of the 3389 Participants in the Survey Study^a^

Characteristic	Participants, No. (%)
Sex	
Male	1627 (48)
Female	1762 (52)
Race	
White	2429 (72)
Black	715 (21)
Other^b^	142 (4)
≥2 Races	103 (3)
Ethnicity	
Non-Hispanic	2599 (77)
Hispanic	790 (23)
Age, y	
18-29	412 (12)
30-44	797 (24)
45-59	956 (28)
≥60	1224 (36)
Household income, $	
≤24 999	444 (13)
25 000-49 999	637 (19)
50 000-99 999	1124 (33)
≥100 000	1184 (35)
Region	
Northeast	549 (16)
Midwest	641 (19)
South	1386 (41)
West	813 (24)
Residence type	
Metropolitan	3016 (89)
Nonmetropolitan	373 (11)
Political ideology	
Liberal	1016 (30)
Moderate	1263 (37)
Conservative	1110 (33)
History of COVID-19	
Yes or maybe	101 (3)
No	3288 (97)

^a^ Table excludes 158 individuals with incomplete responses to the survey questions.

^b^ Other includes Asian, American Indian and Alaska Native, and Hawaiian and Pacific Islander.

### Support for Public Health Digital Data Use Scenarios

**Figure 1.**
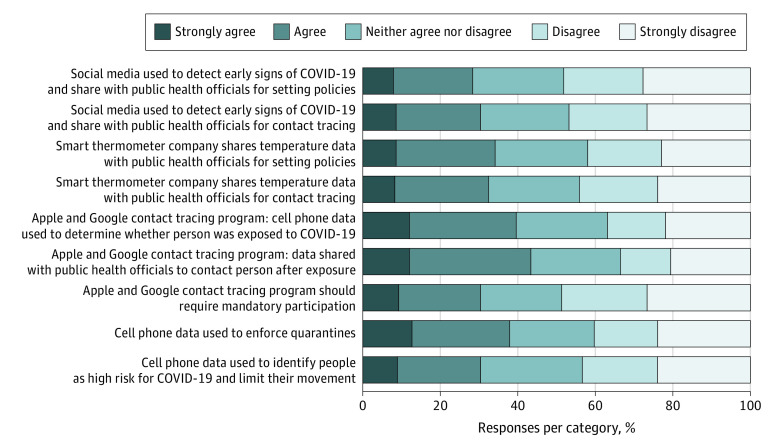
Approval for COVID-19 Digital Data Use Scenarios The weighted distribution of responses to the 9 COVID-19 digital data use scenarios is shown.

Overall, 28% to 43% of the respondents agreed or strongly agreed with any of the digital data use scenarios described for COVID-19 mitigation. Figure 1 shows the response distribution for each of the 9 scenarios. Large political differences were observed in support for the Apple-Google digital contact tracing program, with less support from conservative (coefficient, −0.99; 95% CI, −1.11 to −0.87; *P* < .001) and moderate (coefficient, −0.59; 95% CI, −0.69 to −0.48; *P* < .001) individuals as compared with liberal individuals. Support was greatest for use of smartphones for digital contact tracing with and without public health agency involvement (43% and 40%, respectively) and for quarantine enforcement (38%). Support was lowest for monitoring social media chatter to set public health policies (28%) or contact individuals for testing (31%), making the Apple-Google digital contact tracing program mandatory (30%), and using smartphones to create COVID-19 risk profiles (31%). When agree and neutral responses were combined, the overall proportion ranged from 52% to 67%.

In multivariable models for the 9 scenarios (Table 2), consistent associations were found between self-described political ideology and approval of the scenarios. Compared with liberal individuals, moderate and conservative individuals were less likely to support all 9 scenarios. Personal experience with COVID-19 (ie, personal infection or infection of a family member) and higher local COVID-19 incidence rates were generally not associated with support. Respondents in racial/ethnic minority groups expressed greater support for all the scenarios with the exception of the smart thermometer scenarios and the smartphone scenario that did not involve formal public health contact tracing (no significant differences were found between White and Black respondents for these scenarios). However, compared with White respondents, Black respondents were more supportive of the Apple-Google contact tracing program (coefficient, 0.20; 95% CI, 0.07-0.32; *P* = .002). A consistent association with age across the scenarios was not observed, although older adults (age ≥60 years) expressed greater support than younger adults for the Apple-Google digital contact tracing program (coefficient, 0.28; 95% CI, 0.14-0.43; *P* < .001).

**Table 2.** Factors Associated With Support for 9 Public Health Digital Data Use Scenarios

Factor	Social media				Smart thermometer				Apple and Google program						Smartphones			
	Setting policies		Contact tracing		Setting policies		Contact tracing		Identify exposure		Contact tracing		Mandatory participation		Enforce quarantine		Limit movement	
	Coefficient (95% CI)^a^	*P* value^b^	Coefficient (95% CI)^a^	*P* value^b^	Coefficient (95% CI)^a^	*P* value^b^	Coefficient (95% CI)^a^	*P* value^b^	Coefficient (95% CI)^a^	*P* value^b^	Coefficient (95% CI)^a^	*P* value^b^	Coefficient (95% CI)^a^	*P* value^b^	Coefficient (95% CI)^a^	*P* value^b^	Coefficient (95% CI)^a^	*P* value^b^
Political ideology																		
Liberal	1 [Reference]		1 [Reference]		1 [Reference]		1 [Reference]		1 [Reference]		1 [Reference]		1 [Reference]		1 [Reference]		1 [Reference]	NA
Moderate	−0.40 (−0.51 to −0.29)	<.001	−0.42 (−0.54 to 0.31)	<.001	−0.39 (−0.50 to −0.27)	<.001	−0.35 (−0.46 to −0.24)	<.001	−0.54 (−0.65 to −0.43)	<.001	−0.59 (−0.69 to −0.48)	<.001	−0.42 (−0.53 to −0.30)	<.001	−0.49 (−0.61 to −0.38)	<.001	−0.39 (−0.50 to −0.28)	<.001
Conservative	−0.76 (−0.88 to −0.64)	<.001	−0.78 (−0.90 to −0.66)	<.001	−0.77 (−0.89 to −0.65)	<.001	−0.70 (−0.82 to −0.58)	<.001	−0.95 (−1.10 to −0.83)	<.001	−0.99 (−1.11 to −0.87)	<.001	−0.91 (−1.03 to −0.79)	<.001	−0.97 (−1.10 to −0.85)	<.001	−0.80 (−0.92 to −0.69)	<.001
Prior COVID-19																		
No	−0.12 (−0.40 to 0.16)	.41	−0.03 (−0.30 to 0.24)	.83	0.14 (−0.12 to 0.42)	.31	0.02 (−0.26 to 0.30)	.89	0.02 (−0.27 to 0.30)	.91	−0.03 (−0.32 to 0.26)	.84	−0.03 (−0.32 to 0.26)	.86	0.15 (−0.13 to 0.43)	.29	−0.01 (−0.29 to 0.27)	.97
Race																		
White	1 [Reference]	NA	1 [Reference]	NA	1 [Reference]	NA	1 [Reference]	NA	1 [Reference]	NA	1 [Reference]	NA	1 [Reference]	NA	1 [Reference]	NA	1 [Reference]	NA
Black	0.24 (0.11 to 0.36)	<.001	0.26 (0.13 to 0.38)	<.001	0.07 (−0.05 to 0.19)	.26	0.18 (0.05 to 0.30)	.005	0.07 (−0.06 to 0.19)	.30	0.20 (0.07 to 0.32)	.002	0.32 (0.19 to 0.44)	<.001	0.33 (0.20 to 0.46)	<.001	0.31 (0.19 to 0.44)	<.001
Other^c^	0.58 (0.37 to 0.78)	<.001	0.49 (0.28 to 0.71)	<.001	0.32 (0.12 to 0.52)	.002	0.35 (0.13 to 0.56)	.001	0.47 (0.27 to 0.67)	<.001	0.42 (0.22 to 0.63)	<.001	0.61 (0.39 to 0.82)	<.001	0.73 (0.53 to 0.93)	<.001	0.79 (0.58 to 0.99)	<.001
≥2 Races	0.18 (−0.09 to 0.45)	.19	0.25 (−0.02 to 0.52)	.07	0.18 (−0.09 to 0.44)	.19	0.20 (−0.07 to 0.46)	.15	0.24 (−0.05 to 0.52)	.10	0.26 (−0.02 to 0.53)	.07	0.33 (0.07 to 0.60)	.01	0.23 (−0.04 to 0.50)	.10	0.22 (−0.02 to 0.47)	.07
Ethnicity																		
Hispanic	1 [Reference]	NA	1 [Reference]	NA	1 [Reference]	NA	1 [Reference]	NA	1 [Reference]	NA	1 [Reference]	NA	1 [Reference]	NA	1 [Reference]	NA	1 [Reference]	NA
Non-Hispanic	−0.37 (−0.49 to −0.26)	<.001	−0.37 (−0.48 to −0.25)	<.001	−0.25 (−0.37 to −0.14)	<.001	−0.31 (−0.43 to −0.20)	<.001	−0.30 (−0.42 to −0.19)	<.001	−0.26 (−0.38 to −0.14)	<.001	−0.55 (−0.67 to −0.43)	<.001	−0.54 (−0.66 to −0.42)	<.001	−0.46 (−0.57 to −0.34)	<.001
Household income, $																		
≤24 999	1 [Reference]	NA	1 [Reference]	NA	1 [Reference]	NA	1 [Reference]	NA	1 [Reference]	NA	1 [Reference]	NA	1 [Reference]	NA	1 [Reference]	NA	1 [Reference]	NA
25 000-49 999	−0.04 (−0.21 to 0.13)	.62	−0.01 (−0.18 to 0.16)	.92	−0.03 (−0.19 to 0.13)	.69	−0.07 (−0.23 to 0.10)	.43	−0.07 (−0.24 to 0.09)	.39	0 (−0.16 to 0.17)	.98	−0.06 (−0.22 to 0.10)	.47	−0.10 (−0.27 to 0.07)	.26	0.03 (−0.13 to 0.19)	.70
50 000-99 999	−0.23 (−0.38 to 0.08)	.003	−0.18 (−0.33 to −0.04)	.02	−0.16 (−0.30 to −0.01)	.03	−0.21 (−0.36 to −0.07)	.004	−0.24 (−0.39 to −0.09)	.002	−0.19 (−0.34 to −0.04)	.01	−0.30 (−0.44 to −0.16)	<.001	−0.32 (−0.47 to −0.17)	<.001	−0.21 (−0.35 to −0.06)	.005
≥100 000	−0.05 (−0.20 to 0.10)	.52	−0.01 (−0.16 to 0.14)	.92	−0.1 (−0.25 to 0.05)	.19	−0.15 (−0.29 to 0.00)	.05	−0.05 (−0.20 to 0.10)	.50	−0.06 (−0.21 to 0.09)	.42	−0.14 (−0.29 to 0.00)	.05	−0.20 (−0.35 to −0.05)	.009	−0.10 (−0.25 to 0.04)	.17
Age, y																		
18-29	1 [Reference]	NA	1 [Reference]	NA	1 [Reference]	NA	1 [Reference]	NA	1 [Reference]	NA	1 [Reference]	NA	1 [Reference]	NA	1 [Reference]	NA	1 [Reference]	NA
30-44	0.09 (−0.06 to 0.24)	.24	0.07 (–0.08 to 0.21)	.37	0.03 (−0.12 to 0.18)	.70	0 (−0.15 to 0.16)	.96	0.06 (−0.09 to 0.21)	.45	0.09 (−0.06 to 0.25)	.22	0.01 (−0.15 to 0.16)	.93	0.15 (0.00 to 0.30)	.05	0.10 (−0.05 to 0.24)	.20
45-59	0.01 (−0.14 to 0.15)	.95	0.01 (−0.13 to 0.17)	.80	−0.09 (−0.24 to 0.05)	.22	−0.09 (−0.24 to 0.06)	.24	0.03 (−0.12 to 0.18)	.72	0.09 (−0.06-\to 0.24)	.24	0.04 (−0.11 to 0.19)	.61	0.16 (0.01 to 0.31)	.04	0.06 (−0.08 to 0.21)	.41
≥60	0.06 (−0.08 to 0.21)	.41	0.15 (0.01 to 0.29)	.04	−0.04 (−0.19 to 0.10)	.55	−0.01 (−0.15 to 0.14)	.93	0.14 (−0.01 to 0.28)	.07	0.28 (0.14 to 0.43)	<.001	0.10 (−0.04 to 0.25)	.16	0.29 (0.14 to 0.44)	<.001	0.16 (0.02 to 0.30)	.02
COVID-19 incidence, per 10 000 cases																		
<48	1 [Reference]	NA	1 [Reference]	NA	1 [Reference]	NA	1 [Reference]	NA	1 [Reference]	NA	1 [Reference]	NA	1 [Reference]	NA	1 [Reference]	NA	1 [Reference]	NA
48-95	−0.12 (−0.28 to 0.04)	.13	−0.10 (−0.26 to 0.05)	.18	−0.05 (−0.21 to 0.10)	.50	−0.05 (−0.21 to 0.11)	.51	−0.02 (−0.18 to 0.14)	.80	−0.06 (−0.21 to 0.10)	.48	−0.06 (−0.21 to 0.09)	.41	−0.16 (−0.32 to −0.01)	.04	−0.08 (−0.23 to 0.07)	.32
95-186	−0.19 (−0.35 to −0.02)^b^	.03	−0.20 (−0.37 to −0.04)	.02	−0.21 (−0.38 to −0.35)	.02	−0.18 (−0.35 to 0.00)	.05	−0.16 (−0.33 to 0.01)	.07	−0.15 (−0.32 to 0.02)	.08	−0.07 (−0.23 to 0.09)	.40	−0.24 (−0.41 to −0.07)	.006	−0.16 (−0.32 to 0.01)	.06
>186	−0.02 (−0.20 to 0.15)	.79	−0.10 (−0.27 to 0.07)	.27	−0.06 (−0.24 to 0.12)	.51	−0.04 (−0.22 to 0.14)	.67	0.02 (−0.16 to 0.20)	.85	−0.03 (−0.20 to 0.15)	.77	0.1 (−0.07 to 0.27)	.26	−0.08 (−0.25 to 0.10)	.38	0.06 (−0.11 to 0.24)	.48

Abbreviation: NA, not applicable.

^a^ Coefficients from the model represent differences on a scale of 1 to 5, where 1 indicates strongly disagree and 5, strongly agree; positive coefficients represent greater support. Models were adjusted for the 4 census regions.

^b^ With a Bonferroni correction, *P* < .006 indicates statistical significance.

^c^ Includes Asian, American Indian and Alaska Native, and Hawaiian and Pacific Islander.

### Factors Associated With Overall Support for Public Health Digital Data Use

We also assessed all scenarios together using a single model (Table 3). The model included 3 factors identified through factor analysis, representing the 9 scenarios: cluster 1 (scenarios 1 and 2: social media data), cluster 2 (scenarios 3 and 4: smart thermometer data), and cluster 3 (scenarios 5 and 9: smartphone data). Coefficients from the model represent differences on a scale of 1 to 5, where 1 indicates strongly disagree and 5, strongly agree; positive coefficients represent greater support. Support was greater for smartphone data (coefficient, 0.29; 95% CI, 0.23-0.35; *P* < .001) and smart thermometer data (coefficient, 0.09; 95% CI, 0.03-0.16; *P* = .004) compared with social media data. Second, there were significant differences by political ideology; compared with liberal individuals, support for the scenarios overall was lower among conservative (coefficient, −0.81; 95% CI, −0.96 to −0.66; *P* < .001) and moderate (coefficient, −0.52; 95% CI, −0.67 to −0.38; *P* < .001) individuals. These political differences were consistent among the 3 clusters of scenarios, although conservative individuals were less likely to support use of smartphone data (coefficient, −0.15; 95% CI, −0.23 to −0.07; *P* < .001) than were liberal individuals. Members of racial/ethnic minority groups expressed greater support compared with nonminority respondents (Black respondents vs White respondents: coefficient, 0.22; 95% CI, 0.12-0.32; Hispanic respondents vs non-Hispanic respondents: coefficient, 0.38; 95% CI, 0.28-0.48; *P* < .001 for both). Prior personal or family experience with COVID-19 (coefficient, 0.00; 95% CI, −0.25 to 0.25) and local COVID-19 incidence rates (quartile 4 vs quartile 1: coefficient, −0.02; 95% CI, −0.16 to 0.13) were not associated with support for data use.

**Table 3.** Factor Analysis for COVID-19 Public Health Digital Data Use Scenarios^a^

Factor	Coefficient (95% CI)^a^	*P* value
Political ideology		
Liberal	1 [Reference]	NA
Moderate	−0.52 (−0.67 to −0.38)	<.001
Conservative	−0.81 (−0.96 to −0.66)	<.001
Race		
White	1 [Reference]	NA
Black	0.22 (0.12 to 0.32)	<.001
Other^b^	0.53 (0.36 to 0.70)	<.001
≥2 Races	0.24 (0.01 to 0.47)	.04
Ethnicity		
Non-Hispanic	1 [Reference]	NA
Hispanic	0.38 (0.28 to 0.48)	<.001
Household income, $		
≤24 999	1 [Reference]	NA
25 000-49 999	−0.04 (−0.17 to 0.10)	.61
50 000-99 999	−0.23 (−0.35 to −0.11)	<.001
≥100 000	−0.09 (−0.22 to 0.03)	.14
Region		
Northeast	1 [Reference]	NA
Midwest	−0.06 (−0.21 to 0.09)	.41
South	0.00 (−0.15 to 0.15)	.99
West	0.00 (−0.15 to 0.15)	.98
Age, y		
18-29	1 [Reference]	NA
30-44	0.07 (−0.06 to 0.19)	.30
45-59	0.03 (−0.09 to 0.15)	.65
≥60	0.13 (0.01 to 0.25)	.03
Family history of COVID-19		
Yes	1 [Reference]	NA
No	0.00 (−0.25 to 0.25)	.99
COVID-19 incidence rate		
Quartile 1	1 [Reference]	NA
Quartile 2	−0.08 (−0.21 to 0.05)	.22
Quartile 3	−0.18 (−0.32 to −0.04)	.01
Quartile 4	−0.02 (−0.16 to 0.13)	.80
Residence type		
Nonmetropolitan	1 [Reference]	NA
Metropolitan	0.13 (0.00 to 0.25)	.05
Framing		
Individual level	1 [Reference]	NA
Population level	−0.08 (−0.21 to 0.05)	.21
Factor main effects		
Social media scenarios	1 [Reference]	NA
Smart thermometer scenarios	0.09 (0.03 to 0.16)	.004
Smartphone scenarios	0.29 (0.23 to 0.35)	<.001
Interaction terms		
Population-level framing × political ideology		
Liberal	1 [Reference]	NA
Moderate	0.21 (0.02 to 0.39)	.03
Conservative	0.09 (−0.11 to 0.29)	.38
Smart thermometer × political ideology		
Liberal	1 [Reference]	NA
Moderate	0.03 (−0.05 to 0.12)	.44
Conservative	0.03 (−0.05 to 0.12)	.44
Smartphone × political ideology		
Liberal	1 [Reference]	NA
Moderate	−0.06 (−0.14 to 0.02)	.13
Conservative	−0.15 (−0.23 to −0.07)	<.001

Abbreviation: NA, not applicable.

^a^ Coefficients from the model represent differences on a scale of 1 to 5, where 1 indicates strongly disagree and 5, strongly agree; positive coefficients represent greater support.

^b^ Asian, American Indian and Alaska Native, and Hawaiian and Pacific Islander.

In this model, we also evaluated the effect of the framing experiment embedded in the survey design. There were no statistically significant differences in responses between those randomly assigned to scenarios that were written to frame data use at the individual level vs the population level (“your data” vs “people’s data”). However, some differences were associated with political ideology. Moderates responded more favorably to scenarios that used population-level vs individual-level framing (coefficient, 0.21; 95% CI, 0.02-0.39; *P* = .03).

### Support for the Apple-Google Digital Contact Tracing Program

In our survey, 40% of respondents supported the Apple-Google digital contact tracing program. When we described making data sharing with public health departments a standard part of the program, we observed no decrease in support (43%). However, making the program mandatory led to a decrease in support to 30%.

**Figure 2.**
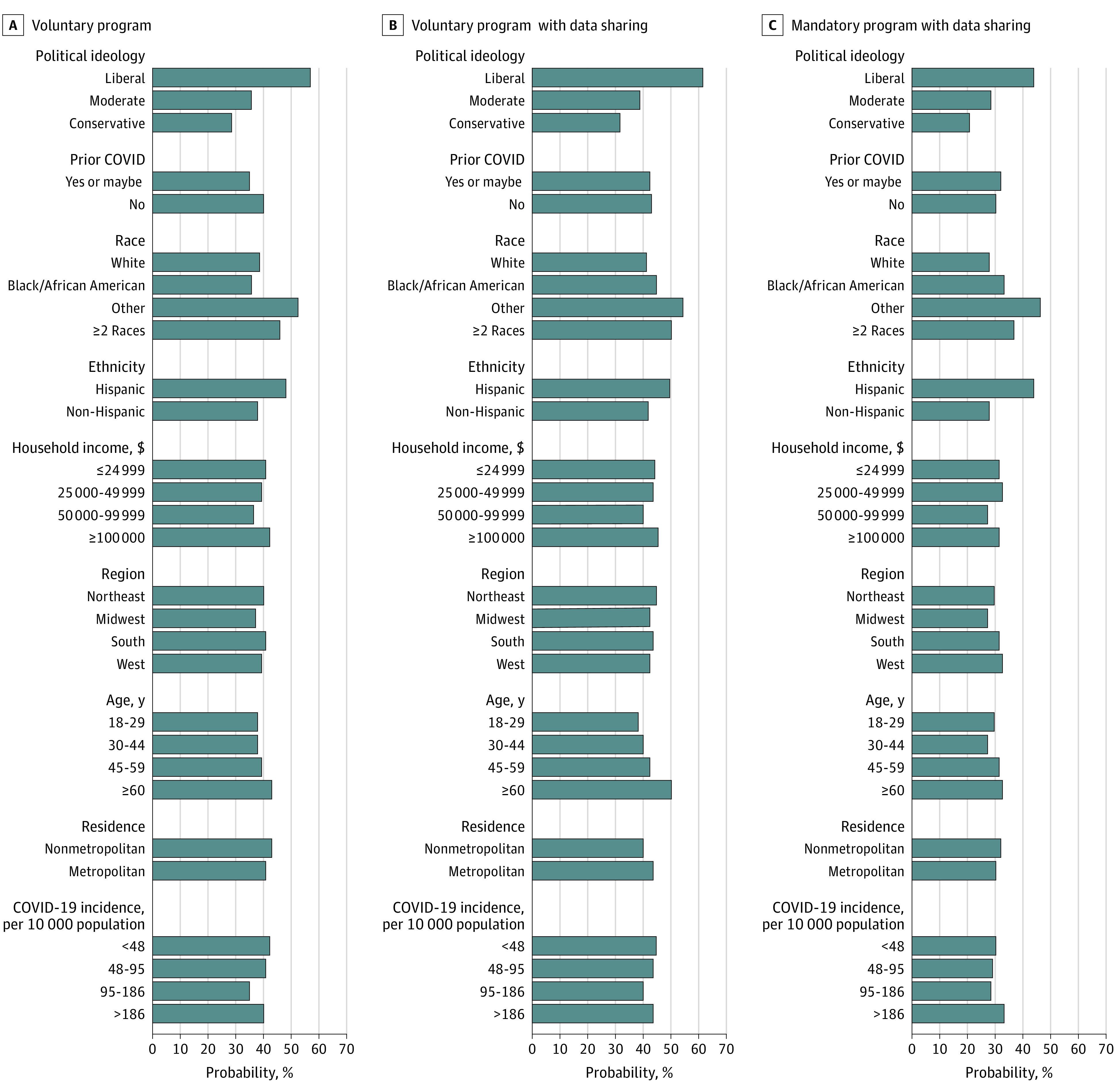
Support for Apple-Google Contact Tracing Program Adjusted probabilities of support for 3 possible variations of the Apple-Google digital contact tracing program are shown.

Figure 2 shows the results for the Apple-Google programs from Table 2 as adjusted probabilities of support. Across the 3 scenarios, liberal individuals were more supportive of the program than conservative individuals. For a completely voluntary program (scenario 5), 57% of liberal individuals supported it compared with 36% of moderates and 29% of conservative individuals. The differences were similar for a voluntary program with public health involvement (scenario 6). Across the 3 scenarios, respondents in racial/ethnic minority groups were more supportive, with differences between Hispanic and non-Hispanic individuals ranging from 7.8 to 16.3 percentage points. Black respondents were more supportive of the program with public health involvement (difference, 3.5 percentage points) and a mandatory program (difference, 5.1 percentage points) compared with White respondents.

## Discussion

Although digital technologies offer substantial opportunities to mitigate the effects of pandemics on public health, they also invite questions about balancing individual privacy and public health benefits. There were 3 key findings of this survey study. First, consumers reported reluctance to give up digital privacy for public health benefit.^28^ None of the scenarios we presented received majority support: the highest level of support was 43% for using smartphones for contact tracing. Public health departments across the nation are deploying the Apple-Google Bluetooth-enabled contact tracing program. Our findings suggest that well-designed initiatives are needed to achieve widespread adoption. Given the large percentage of respondents who provided neutral responses (range, 23.2%-26.3%), adoption by employers or educational institutions might be a strategy to increase uptake along with opting out or other behavioral nudges to encourage use; people may be amenable to influence.^29,30^

Second, personal experience with COVID-19 or living in a county with higher COVID-19 rates was not associated with support for public health digital tools. Instead, political ideology was the greatest contributor to variation in consumer views; conservative individuals were less supportive of using digital tools to mitigate the transmission of COVID-19 than were moderate and liberal individuals. Prior studies in which consumer views on digital privacy were examined revealed conflicting associations with political ideology, perhaps because of views being issue dependent.^31,32,33^ For example, conservative individuals have been more supportive than liberal individuals of requiring Apple to make information available to law enforcement agencies to solve crimes and allowing government surveillance to monitor compliance with government programs.^31,32,33^ However, other surveys have revealed increasing privacy concerns among conservative individuals and reduced concerns among liberal individuals.^32^ Our findings related to public health digital privacy may have been associated with the degree to which COVID-19 became a partisan issue in the 2020 election season.^34,35^

Third, respondents from racial/ethnic minority groups expressed greater support for use of digital data to address the pandemic than did White and non-Hispanic respondents. This association was consistent in nearly all 9 scenarios and in a model in which all scenarios were combined. Results of prior research^36^ suggest that Black and Hispanic adults have higher levels of concern about online privacy and security than do White adults. However, Black and Hispanic adults have also reported greater trust in health information from the internet compared with White respondents^37^ and report feeling more in control of their online information than White adults.^38^ The higher levels of support for these public health digital tools among respondents from racial/ethnic minority groups may be explained by the disproportionate health effects COVID-19 has had in these populations.

Our findings suggest that many US adult consumers may be reluctant to give up digital privacy even when facing substantial health and economic harm associated with the COVID-19 pandemic. Politicization of the pandemic may have contributed to these privacy concerns because conservative individuals were less supportive of these digital public health tools. Despite these political differences, many liberal individuals also expressed reticence. These concerns are not consistent with the nearly ubiquitous sharing of digital data in commercial settings, providing arguably less individual or social benefit.^38^ However, most commercial data sharing occurs not because consumers are willingly sharing but because it has become difficult to avoid sharing.^39,40^

The Apple-Google Bluetooth-enabled contact tracing program was designed with strong privacy protections, generally exceeding those found in most digital devices and programs in the commercial marketplace.^41,42^ The challenge for the public health community to deploy these tools has been to convince a wary public that privacy will be maintained. Achieving widespread adoption may require intensive advertising, trusted messengers, and behavioral nudges to encourage adoption. For example, results from a previous study^43^ suggest that successes are associated with use of trusted messengers to overcome issues of general mistrust and partisan differences in public health concerns. Without these efforts, our data suggest that most of the population will not seek out these programs and opt in.

### Limitations

This study has limitations. The results of this survey are reflective of the time during which it was administered, from July 10 to July 31, 2020. Attitudes toward COVID-19 control may have changed as COVID-19 rates changed around the country. In addition, as with all survey findings, there may be important differences in attitudes or views between responders and nonresponders, although our survey had a relatively high response rate. We asked consumers about scenarios that many believe are hypothetical. We might have observed different responses if we had presented individuals with an actual program and asked them to consent to participate.

## Conclusions

In this survey study of US adults, many US adults were averse to their information being used on digital platforms to mitigate transmission of COVID-19. These findings suggest that in current and future pandemics, public health departments should use multiple strategies to gain public trust and accelerate adoption of tools such as digital contact tracing applications.

## Notes

Supplement. **eAppendix 1.** Summary of Scenarios **eAppendix 2.** Comparison of Results With Multiple Imputation: Social Media and Smart Thermometer **eAppendix 3.** Comparison of Results With Multiple Imputation: Apple and Google Program and Smart Phones
