# Supplementary material for: Consumer Views on Using Digital Data for COVID-19 Control in the United States
Source: JAMA Netw Open. 2021 May 19;4(5):e2110918. doi: 10.1001/jamanetworkopen.2021.10918 (PMC8134997; doi:10.1001/jamanetworkopen.2021.10918)
Supplement: Supplement. — eAppendix 1. Summary of Scenarios eAppendix 2. Comparison of Results With Multiple Imputation: Social Media and Smart Thermometer eAppendix 3. Comparison of Results With Multiple Imputation: Apple and Google Program and Smart Phones [file jamanetwopen-e2110918-s001.pdf]

Supplementary Online Content

Grande D, Mitra N, Marti XL, et al. Consumer views on using digital data for COVID-19 control in the United States. *JAMA Netw Open*. 2021;4(5):e2110918. doi:10.1001/jamanetworkopen.2021.10918

eAppendix 1. Summary of Scenarios

eAppendix 2. Comparison of Results With Multiple Imputation: Social Media and Smart Thermometer

eAppendix 3. Comparison of Results With Multiple Imputation: Apple and Google Program and Smart Phones

This supplementary material has been provided by the authors to give readers additional information about their work.

© 2021 Grande D et al. *JAMA Network Open*.

eAppendix 1. Summary of Scenarios

	Summary
1	Social media used to detect early signs of COVID-19 and share with public health officials for setting policies
2	Social media used to detect early signs of COVID-19 and share with public health officials for contact tracing
3	Smart thermometer company shares temperature data with public health officials for setting policies
4	Smart thermometer company shares temperature data with public health officials for contact tracing
5	Apple & Google contact tracing program: cell phone data used to determine if you were exposed to COVID-19
6	Apple & Google contact tracing program: data shared with public health officials to contact you after exposure
7	Apple & Google contact tracing program should require mandatory participation
8	Cell phone data used to enforce quarantines
9	Cell phone data used to identify people as high risk for COVID-19 and limit their movement

© 2021 Grande D et al. *JAMA Network Open*.

eAppendix 2. Comparison of results with multiple imputation

	Social Media						Smart Thermometer					
	Setting Policies			Contact Tracing			Setting Policies			Contact Tracing		
	Multiple Imputation	Complete Cases	Extreme Cases	Multiple Imputation	Complete Cases	Extreme Cases	Multiple Imputation	Complete Cases	Extreme Cases	Multiple Imputation	Complete Cases	Extreme Cases
	Coefficient<br>95% CI<br>p value	Coefficient<br>95% CI<br>p value	Coefficient<br>95% CI<br>p value	Coefficient<br>95% CI<br>p value	Coefficient<br>95% CI<br>p value	Coefficient<br>95% CI<br>p value	Coefficient<br>95% CI<br>p value	Coefficient<br>95% CI<br>p value	Coefficient<br>95% CI<br>p value	Coefficient<br>95% CI<br>p value	Coefficient<br>95% CI<br>p value	Coefficient<br>95% CI<br>p value
**Political Ideology**												
Liberal	Reference											
Moderate	-0.40<br>-0.51, -0.29<br><0.001	-0.39<br>-0.51, -0.28<br><0.001	-0.40<br>-0.52, -0.29<br><0.001	-0.42<br>-0.54, 0.31<br><0.001	-0.42<br>-0.54, -0.30<br><0.001	-0.42<br>-0.54, -0.30<br><0.001	-0.39<br>-0.50, -0.27<br><0.001	-0.38<br>-0.49, -0.27<br><0.001	-0.38<br>-0.50, -0.27<br><0.001	-0.35<br>-0.46, -0.24<br><0.001	-0.35<br>-0.46, -0.23<br><0.001	-0.35<br>-0.46, -0.24<br><0.001
Conservative	-0.76<br>-0.88, -0.64<br><0.001	-0.76<br>-0.88, -0.64<br><0.001	-0.73<br>-0.85, -0.61<br><0.001	-0.78<br>-0.90, -0.66<br><0.001	-0.79<br>-0.92, -0.67<br><0.001	-0.76<br>-0.88, -0.64<br><0.001	-0.77<br>-0.89, -0.65<br><0.001	-0.77<br>-0.89, -0.65<br><0.001	-0.74<br>-0.86, -0.62<br><0.001	-0.70<br>-0.82, -0.58<br><0.001	-0.70<br>-0.83, -0.58<br><0.001	-0.66<br>-0.78, -0.54<br><0.001
**Prior COVID-19**												
No	-0.12<br>-0.40, 0.16<br>0.41	-0.07<br>-0.35, 0.22<br>0.65	-0.10<br>-0.38, 0.18<br>0.49	-0.03<br>-0.30, 0.24<br>0.83	-0.01<br>-0.30, 0.26<br>0.92	-0.01<br>-0.28, 0.26<br>0.93	0.14<br>-0.12, 0.42<br>0.31	0.16<br>-0.12, 0.44<br>0.27	0.17<br>-0.10, 0.45<br>0.22	0.02<br>-0.26, 0.30<br>0.89	0.06<br>-0.22, 0.34<br>0.68	0.04<br>-0.24, 0.32<br>0.77
**Race**												
White	Reference	Reference	Reference									
Black/African American	0.24<br>0.11, 0.36<br><0.001	0.24<br>0.11, 0.37<br><0.001	0.24<br>0.12, 0.37<br><0.001	0.26<br>0.13, 0.38<br><0.001	0.26<br>0.13, 0.39<br><0.001	0.27<br>0.15, 0.40<br><0.001	0.07<br>-0.05, 0.19<br>0.26	0.09<br>-0.03, 0.22<br>0.14	0.08<br>-0.04, 0.20<br>0.19	0.18<br>0.05, 0.30<br>0.005	0.20<br>0.07, 0.33<br>0.002	0.20<br>0.08, 0.32<br>0.002
Other	0.58<br>0.37, 0.78<br><0.001	0.57<br>0.36, 0.78<br><0.001	0.56<br>0.36, 0.76<br><0.001	0.49<br>0.28, 0.71<br><0.001	0.47<br>0.26, 0.69<br><0.001	0.49<br>0.28, 0.70<br><0.001	0.32<br>0.12, 0.52<br>0.002	0.31<br>0.10, 0.51<br>0.003	0.32<br>0.11, 0.52<br>0.002	0.35<br>0.13, 0.56<br>0.001	0.34<br>0.12, 0.56<br>0.002	0.35<br>0.13, 0.56<br>0.002
2+ Races	0.18<br>-0.09, 0.45<br>0.19	0.21<br>-0.07, 0.49<br>0.15	0.20<br>-0.07, 0.46<br>0.15	0.25<br>-0.02, 0.52<br>0.07	0.27<br>-0.02, 0.55<br>0.06	0.27<br>-0.01, 0.53<br>0.06	0.18<br>-0.09, 0.44<br>0.19	0.19<br>-0.08, 0.47<br>0.17	0.20<br>-0.07, 0.46<br>0.14	0.2<br>-0.07, 0.46<br>0.15	0.24<br>-0.04, 0.52<br>0.10	0.21<br>-0.05, 0.48<br>0.12
**Ethnicity**												
Hispanic	Reference											
Non-Hispanic	-0.37<br>-0.49, -0.26<br><0.001	-0.37<br>-0.48, -0.25<br><0.001	-0.37<br>-0.49, -0.25<br><0.001	-0.37<br>-0.48, -0.25<br><0.001	-0.35<br>-0.47, -0.23<br><0.001	-0.37<br>-0.49, -0.25<br><0.001	-0.25<br>-0.37, -0.14<br><0.001	-0.27<br>-0.39, -0.15<br><0.001	-0.25<br>-0.37, -0.14<br><0.001	-0.31<br>-0.43, -0.20<br><0.001	-0.31<br>-0.43, -0.20<br><0.001	-0.32<br>-0.44, -0.20<br><0.001
**Household Income**												
<$24,999	Reference											
$25,000-$49,999	-0.04<br>-0.21, 0.13<br>0.62	-0.03<br>-0.20, 0.15<br>0.76	-0.06<br>-0.22, 0.11<br>0.52	-0.01<br>-0.18, 0.16<br>0.92	0.02<br>-0.15, 0.19<br>0.82	-0.02<br>-0.19, 0.15<br>0.82	-0.03<br>-0.19, 0.13<br>0.69	-0.01<br>-0.17, 0.16<br>0.93	-0.06<br>-0.22, 0.10<br>0.47	-0.07<br>-0.23, 0.10<br>0.43	-0.04<br>-0.20, 0.13<br>0.66	-0.08<br>-0.25, 0.08<br>0.32
$50,000-$99,999	-0.23<br>-0.38, 0.08<br>0.003	-0.20<br>-0.35, 0.04<br>0.01	-0.23<br>-0.39, -0.08<br>0.003	-0.18<br>-0.33, -0.04<br>0.02	-0.15<br>-0.30, 0.00<br>0.06	-0.19<br>-0.34, 0.03<br>0.02	-0.16<br>-0.30, -0.01<br>0.03	-0.14<br>-0.28, -0.01<br>0.07	-0.18<br>-0.33, -0.03<br>0.02	-0.21<br>-0.36, -0.07<br>0.004	-0.19<br>-0.34, -0.04<br>0.01	-0.23<br>-0.38, -0.08<br>0.002
>$100,000	-0.05<br>-0.20, 0.10<br>0.52	-0.02<br>-0.18, 0.13<br>0.80	-0.06<br>-0.22, 0.09<br>0.42	-0.01<br>-0.16, 0.14<br>0.92	0.02<br>-0.13, 0.17<br>0.81	0.02<br>-0.73, 0.13<br>0.77	-0.1<br>-0.25, 0.05<br>0.19	-0.08<br>-0.23, 0.07<br>0.32	-0.13<br>-0.28, 0.02<br>0.09	-0.15<br>-0.29, 0.00<br>0.05	-0.12<br>-0.27, 0.03<br>0.12	-0.16<br>-0.31, 0.01<br>0.03
**Age**												
18-29	Reference	Reference	Reference									
30-44	0.09<br>-0.06, 0.24<br>0.24	0.09<br>-0.07, 0.24<br>0.27	0.10<br>-0.05, 0.25<br>0.21	0.07<br>-0.08, 0.21<br>0.37	0.07<br>-0.84, 0.22<br>0.39	0.08<br>-0.07, 0.23<br>0.29	0.03<br>-0.12, 0.18<br>0.70	0.03<br>-0.12, 0.18<br>0.67	0.05<br>-0.10, 0.20<br>0.55	0<br>-0.15, 0.16<br>0.96	0<br>-0.16, 0.15<br>0.99	0.02<br>-0.14, 0.17<br>0.85
45-59	0.01<br>-0.14, 0.15<br>0.95	0<br>-0.15, 0.15<br>0.98	-0.01<br>-0.16, 0.14<br>0.89	0.01<br>-0.13, 0.17<br>0.80	0.02<br>-0.13, 0.17<br>0.79	0<br>-0.15, 0.15<br>0.98	-0.09<br>-0.24, 0.05<br>0.22	-0.08<br>-0.23, 0.07<br>0.28	-0.1<br>-0.25, 0.05<br>0.18	-0.09<br>-0.24, 0.06<br>0.24	-0.09<br>-0.24, 0.07<br>0.26	-0.10<br>-0.25, 0.05<br>0.20
60+	0.06<br>-0.08, 0.21<br>0.41	0.07<br>-0.08, 0.22<br>0.37	0.05<br>-0.09, 0.20<br>0.49	0.15<br>0.01, 0.29<br>0.04	0.15<br>0.01, 0.30<br>0.04	0.14<br>-0.01, 0.28<br>0.06	-0.04<br>-0.19, 0.10<br>0.55	-0.03<br>-0.17, 0.12<br>0.71	-0.01<br>-0.20, 0.01<br>0.45	-0.01<br>-0.15, 0.14<br>0.93	0<br>-0.15, 0.15<br>0.96	-0.01<br>-0.16, 0.13<br>0.85
**COVID-19 Incidence**												
<48 per 10,000 Cases	Reference	Reference	Reference									
48-95 per 10,000 Cases	-0.12<br>-0.28, 0.04<br>0.13	-0.10<br>-0.26, 0.05<br>0.20	-0.13<br>-0.28, 0.03<br>0.10	-0.10<br>-0.26, 0.05<br>0.18	-0.08<br>-0.24, 0.07<br>0.30	-0.12<br>-0.28, 0.03<br>0.12	-0.05<br>-0.21, 0.10<br>0.50	-0.03<br>-0.19, 0.13<br>0.69	-0.03<br>-0.19, 0.13<br>0.69	-0.05<br>-0.21, 0.11<br>0.51	-0.05<br>-0.21, 0.12<br>0.58	-0.05<br>-0.21, 0.11<br>0.55
95-186 per 10,000 Cases	-0.19<br>-0.35, -0.02<br>0.03	-0.17<br>-0.34, -0.03<br>0.06	-0.20<br>-0.37, -0.04<br>0.02	-0.20<br>-0.37, -0.04<br>0.02	-0.17<br>-0.34, 0.00<br>0.05	-0.22<br>-0.39, -0.06<br>0.009	-0.21<br>-0.38, -0.35<br>0.02	-0.17<br>-0.35, 0.00<br>0.05	-0.20<br>-0.37, 0.03<br>0.03	-0.18<br>-0.35,0.00<br>0.05	-0.15<br>-0.33, 0.03<br>0.09	-0.17<br>-0.34, 0.00<br>0.06
>186 per 10,000 Cases	-0.02<br>-0.20, 0.15<br>0.79	-0.01<br>-0.19, 0.16<br>0.90	-0.02<br>-0.19, 0.14<br>0.78	-0.10<br>-0.27, 0.07<br>0.27	-0.08<br>-0.25, 0.09<br>0.37	-0.11<br>-0.27, 0.06<br>0.21	-0.06<br>-0.24, 0.12<br>0.51	-0.05<br>-0.24, 0.13<br>0.56	-0.03<br>-0.21, 0.14<br>0.70	-0.04<br>-0.22, 0.14<br>0.67	-0.05<br>-0.23, 0.14<br>0.63	-0.02<br>-0.20, 0.16<br>0.81

© 2021 Grande D et al. *JAMA Network Open*.

eAppendix 3. Comparison of results with multiple imputation

	Apple & Google Program								
	Identify Exposure			Contact Tracing			Mandatory Participation		
	Multiple Imputation	Complete Cases	Extreme Cases	Multiple Imputation	Complete Cases	Extreme Cases	Multiple Imputation	Complete Cases	Extreme Cases
	Coefficient<br>95% CI<br>p value	Coefficient<br>95% CI<br>p value	Coefficient<br>95% CI<br>p value	Coefficient<br>95% CI<br>p value	Coefficient<br>95% CI<br>p value	Coefficient<br>95% CI<br>p value	Coefficient<br>95% CI<br>p value	Coefficient<br>95% CI<br>p value	Coefficient<br>95% CI<br>p value
**Political Ideology**									
Liberal	Reference								
Moderate	-0.54<br>-0.65, -0.43<br><0.001	-0.54<br>-0.65, -0.43<br><0.001	-0.53<br>-0.64, -0.42<br><0.001	-0.59<br>-0.69, -0.48<br><0.001	-0.59<br>-0.70, -0.48<br><0.001	-0.59<br>-0.70, -0.48<br><0.001	-0.42<br>-0.53, -0.30<br><0.001	-0.40<br>-0.52, -0.29<br><0.001	-0.42<br>-0.53, -0.31<br><0.001
Conservative	-0.95<br>-1.10, -0.83<br><0.001	-0.97<br>-1.10, -0.84<br><0.001	-0.91<br>-1.03, -0.79<br><0.001	-0.99<br>-1.11, -0.87<br><0.001	-0.10<br>-1.13, -0.87<br><0.001	-0.96<br>-1.08, -0.85<br><0.001	-0.91<br>-1.03, -0.79<br><0.001	-0.91<br>-1.02, -0.79<br><0.001	-0.87<br>-0.98, -0.75<br><0.001
**Prior COVID-19**									
No	0.02<br>-0.27, 0.30<br>0.91	0.07<br>-0.22, 0.37<br>0.62	0.05<br>-0.24, 0.33<br>0.75	-0.03<br>-0.32, 0.26<br>0.84	-0.03<br>-0.27, 0.33<br>0.86	-0.01<br>-0.29, 0.30<br>0.99	-0.03<br>-0.32, 0.26<br>0.86	0.01<br>-0.29, 0.31<br>0.93	0.01<br>-0.31, 0.28<br>0.91
**Race**									
White	Reference								
Black/African American	0.07<br>-0.06, 0.19<br>0.30	0.08<br>-0.05, 0.21<br>0.23	0.09<br>-0.04, 0.22<br>0.17	0.20<br>0.07, 0.32<br>0.002	0.22<br>0.09, 0.34<br>0.001	0.20<br>0.08, 0.33<br>0.001	0.32<br>0.19, 0.44<br><0.001	0.34<br>0.22, 0.47<br><0.001	0.34<br>0.22, 0.46<br><0.001
Other	0.47<br>0.27, 0.67<br><0.001	0.48<br>0.27, 0.68<br><0.001	0.50<br>0.30, 0.70<br><0.001	0.42<br>0.22, 0.63<br><0.001	0.45<br>0.24, 0.65<br><0.001	0.42<br>0.22, 0.62<br><0.001	0.61<br>0.39, 0.82<br><0.001	0.64<br>0.42, 0.86<br><0.001	0.60<br>0.38, 0.81<br><0.001
2+ Races	0.24<br>-0.05, 0.52<br>0.10	0.26<br>-0.04, 0.55<br>0.09	0.26<br>-0.03, 0.55<br>0.08	0.26<br>-0.02, 0.53<br>0.07	0.30<br>-0.01, 0.59<br>0.04	0.27<br>-0.03, 0.54<br>0.05	0.33<br>0.07, 0.60<br>0.01	0.37<br>0.10, 0.65<br>0.008	0.35<br>0.08, 0.61<br>0.01
**Ethnicity**									
Hispanic	Reference								
Non-Hispanic	-0.30<br>-0.42, -0.19<br><0.001	-0.30<br>-0.42, -0.18<br><0.001	-0.31<br>-0.43, -0.19<br><0.001	-0.26<br>-0.38, -0.14<br><0.001	-0.26<br>-0.38, -0.14<br><0.001	-0.25<br>-0.37, -0.13<br><0.001	-0.55<br>-0.67, -0.43<br><0.001	-0.56<br>-0.68, -0.44<br><0.001	-0.57<br>-0.69, -0.45<br><0.001
**Household Income**									
<$24,999	Reference								
$25,000-$49,999	-0.07<br>-0.24, 0.09<br>0.39	-0.06<br>-0.23, 0.11<br>0.51	-0.09<br>-0.26, 0.08<br>0.29	0<br>-0.16, 0.17<br>0.98	0.03<br>-0.14, 0.20<br>0.75	0.01<br>-0.17, 0.16<br>0.95	-0.06<br>-0.22, 0.10<br>0.47	-0.03<br>-0.19, 0.13<br>0.70	-0.08<br>-0.24, 0.08<br>0.33
$50,000-$99,999	-0.24<br>-0.39, -0.09<br>0.002	-0.20<br>-0.36, -0.05<br>0.01	-0.25<br>-0.40, -0.10<br>0.001	-0.19<br>-0.34, -0.04<br>0.01	-0.16<br>-0.31, 0.00<br>0.04	-0.19<br>-0.35, -0.04<br>0.01	-0.30<br>-0.44, -0.16<br><0.001	-0.28<br>-0.42, -0.13<br><0.001	-0.32<br>-0.47, -0.18<br><0.001
>$100,000	-0.05<br>-0.20, 0.10<br>0.50	-0.01<br>-0.17, 0.14<br>0.86	-0.07<br>-0.22, 0.08<br>0.37	-0.06<br>-0.21, 0.09<br>0.42	-0.03<br>-0.18, 0.13<br>0.72	-0.07<br>-0.23, 0.08<br>0.34	-0.14<br>-0.29, 0.00<br>0.05	-0.11<br>-0.26, 0.04<br>0.14	-0.17<br>-0.31, -0.02<br>0.02
**Age**									
18-29	Reference								
30-44	0.06<br>-0.09, 0.21<br>0.45	0.06<br>-0.10, 0.21<br>0.47	0.07<br>-0.08, 0.23<br>0.34	0.09<br>-0.06, 0.25<br>0.22	0.09<br>-0.06, 0.25<br>0.24	0.11<br>-0.04, 0.26<br>0.16	0.01<br>-0.15, 0.16<br>0.93	0<br>-0.15, 0.16<br>0.97	0.01<br>-0.14, 0.16<br>0.88
45-59	0.03<br>-0.12, 0.18<br>0.72	0.03<br>-0.12, 0.18<br>0.70	0.02<br>-0.13, 0.17<br>0.79	0.09<br>-0.06, 0.24<br>0.24	0.10<br>-0.05, 0.25<br>0.21	0.09<br>-0.06, 0.24<br>0.26	0.04<br>-0.11, 0.19<br>0.61	0.04<br>-0.12, 0.19<br>0.64	0.03<br>-0.12, 0.18<br>0.70
60+	0.14<br>-0.01, 0.28<br>0.07	0.14<br>-0.01, 0.29<br>0.06	0.12<br>-0.02, 0.27<br>0.10	0.28<br>0.14, 0.43<br><0.001	0.29<br>0.14, 0.44<br><0.001	0.26<br>0.11, 0.41<br>0.001	0.10<br>-0.04, 0.25<br>0.16	0.11<br>-0.04, 0.26<br>0.15	0.09<br>-0.06, 0.23<br>0.25
**COVID-19 Incidence**									
<48 per 10,000 Cases	Reference								
48-95 per 10,000 Cases	-0.02<br>-0.18, 0.14<br>0.80	0.00<br>-0.16, 0.16<br>0.10	-0.02<br>-0.17, 0.14<br>0.84	-0.06<br>-0.21, 0.10<br>0.48	-0.04<br>-0.19, 0.12<br>0.66	-0.04<br>-0.19, 0.11<br>0.61	-0.06<br>-0.21, 0.09<br>0.41	-0.06<br>-0.21, 0.10<br>0.46	-0.08<br>-0.23, 0.07<br>0.31
95-186 per 10,000 Cases	-0.16<br>-0.33, 0.01<br>0.07	-0.12<br>-0.30, 0.01<br>0.18	-0.16<br>-0.33, 0.01<br>0.07	-0.15<br>-0.32, 0.02<br>0.08	-0.13<br>-0.30, 0.04<br>0.14	-0.14<br>-0.31, 0.03<br>0.10	-0.07<br>-0.23, 0.09<br>0.40	-0.05<br>-0.22, 0.11<br>0.52	-0.09<br>-0.25, 0.07<br>0.28
>186 per 10,000 Cases	0.02<br>-0.16, 0.20<br>0.85	0.04<br>-0.14, 0.23<br>0.65	0.03<br>-0.14, 0.21<br>0.70	-0.03<br>-0.20, 0.15<br>0.77	-0.02<br>-0.20, 0.16<br>0.84	0.00<br>-0.17, 0.17<br>1.0	0.1<br>-0.07, 0.27<br>0.26	0.10<br>-0.08, 0.27<br>0.28	0.08<br>-0.08, 0.25<br>0.33

	Smart Phones					
	Enforce Quarantines			Limit Movement		
	Multiple Imputation	Complete Cases	Extreme Cases	Multiple Imputation	Complete Cases	Extreme Cases
	Coefficient<br>95% CI<br>p value	Coefficient<br>95% CI<br>p value	Coefficient<br>95% CI<br>p value	Coefficient<br>95% CI<br>p value	Coefficient<br>95% CI<br>p value	Coefficient<br>95% CI<br>p value
**Political Ideology**						
Liberal						
Moderate	-0.49<br>-0.61, -0.38<br><0.001	-0.48<br>-0.60, -0.37<br><0.001	-0.50<br>-0.61, -0.39<br><0.001	-0.39<br>-0.50, -0.28<br><0.001	-0.38<br>-0.49, -0.27<br><0.001	-0.38<br>-0.50, -0.27<br><0.001
Conservative	-0.97<br>-1.10, -0.85<br><0.001	-0.97<br>-1.09, -0.85<br><0.001	-0.93<br>-1.05, -0.81<br><0.001	-0.80<br>-0.92, -0.69<br><0.001	-0.80<br>-0.92, -0.68<br><0.001	-0.75<br>-0.86, -0.63<br><0.001
**Prior COVID-19**						
No	0.15<br>-0.13, 0.43<br>0.29	0.21<br>-0.07, 0.49<br>0.15	0.18<br>-0.10, 0.46<br>0.21	-0.01<br>-0.29, 0.27<br>0.97	-0.05<br>-0.24, 0.33<br>0.75	0.02<br>-0.26, 0.30<br>0.88
**Race**						
White						
Black/African American	0.33<br>0.20, 0.46<br><0.001	0.33<br>0.20, 0.46<br><0.001	0.35<br>0.22, 0.49<br><0.001	0.31<br>0.19, 0.44<br><0.001	0.31<br>0.18, 0.44<br><0.001	0.33<br>0.21, 0.46<br><0.001
Other	0.73<br>0.53, 0.93<br><0.001	0.73<br>0.52, 0.94<br><0.001	0.73<br>0.53, 0.94<br><0.001	0.79<br>0.58, 0.99<br><0.001	0.78<br>0.57, 0.99<br><0.001	080<br>0.60, 1.00<br><0.001
2+ Races	0.23<br>-0.04, 0.50<br>0.10	0.26<br>-0.02, 0.54<br>0.07	0.23<br>-0.04, 0.50<br>0.09	0.22<br>-0.02, 0.47<br>0.07	0.26<br>0.00, 0.51<br>0.05	0.26<br>0.01, 0.50<br>0.04
**Ethnicity**						
Hispanic						
Non-Hispanic	-0.54<br>-0.66, -0.42<br><0.001	-0.53<br>-0.65, -0.41<br><0.001	-0.55<br>-0.67, -0.43<br><0.001	-0.46<br>-0.57, -0.34<br><0.001	-0.45<br>-0.57, -0.33<br><0.001	-0.46<br>-0.58, -0.35<br><0.001
**Household Income**						
<$24,999						
$25,000-$49,999	-0.10<br>-0.27, 0.07<br>0.26	-0.08<br>-0.26, 0.09<br>0.35	-0.10<br>-0.27, 0.07<br>0.26	0.03<br>-0.13, 0.19<br>0.70	0.05<br>-0.11, 0.21<br>0.55	0.01<br>-0.15, 0.17<br>0.8
$50,000-$99,999	-0.32<br>-0.47, -0.17<br><0.001	-0.31<br>-0.46, -0.15<br><0.001	-0.34<br>-0.49, -0.19<br><0.001	-0.21<br>-0.35, -0.06<br>0.005	-0.19<br>-0.34, -0.05<br>0.009	-0.23<br>-0.38, -0.08<br>0.002
>$100,000	-0.20<br>-0.35, -0.05<br>0.009	-0.19<br>-0.34, -0.03<br>0.02	-0.22<br>-0.37, -0.07<br>0.004	-0.10<br>-0.25, 0.04<br>0.17	-0.09<br>-0.24, 0.06<br>0.25	-0.12<br>-0.27, 0.03<br>0.11
**Age**						
18-29						
30-44	0.15<br>0.00, 0.30<br>0.05	0.15<br>0.00, 0.31<br>0.05	0.15<br>-0.01, 0.31<br>0.06	0.10<br>-0.05, 0.24<br>0.20	0.10<br>-0.05, 0.25<br>0.21	0.11<br>-0.40, 0.26<br>0.15
45-59	0.16<br>0.01, 0.31<br>0.04	0.16<br>0.01, 0.32<br>0.04	0.15<br>0.00, 0.30<br>0.06	0.06<br>-0.08, 0.21<br>0.41	0.07<br>-0.08, 0.22<br>0.37	0.05<br>-0.10, 0.20<br>0.52
60+	0.29<br>0.14, 0.44<br><0.001	0.30<br>0.15, 0.45<br><0.001	0.26<br>0.11, 0.41<br>0.001	0.16<br>0.02, 0.30<br>0.02	0.17<br>0.03, 0.31<br>0.02	0.14<br>0.00, 0.28<br>0.05
**COVID-19 Incidence**						
<48 per 10,000 Cases						
48-95 per 10,000 Cases	-0.16<br>-0.32, -0.01<br>0.04	-0.14<br>-0.30, -0.02<br>0.08	-0.18<br>-0.33, -0.02<br>0.03	-0.08<br>-0.23, 0.07<br>0.32	-0.04<br>-0.20, 0.11<br>0.58	-0.09<br>-0.25, 0.06<br>0.24
95-186 per 10,000 Cases	-0.24<br>-0.41, -0.07<br>0.006	-0.20<br>-0.37, -0.03<br>0.02	-0.25<br>-0.42, -0.09<br>0.003	-0.16<br>-0.32, 0.01<br>0.06	-0.11<br>-0.28, 0.06<br>0.19	-0.16<br>-0.33, 0.00<br>0.05
>186 per 10,000 Cases	-0.08<br>-0.25, 0.10<br>0.38	-0.06<br>-0.24, 0.12<br>0.50	-0.08<br>-0.25, 0.09<br>0.35	0.06<br>-0.11, 0.24<br>0.48	0.09<br>-0.08, 0.27<br>0.30	0.06<br>-0.11, 0.24<br>0.48

© 2021 Grande D et al. *JAMA Network Open*.
